# Mesenchymal Stem/Stromal Cell-Mediated Mitochondrial Transfer and the Therapeutic Potential in Treatment of Neurological Diseases

**DOI:** 10.1155/2020/8838046

**Published:** 2020-07-07

**Authors:** Deqiang Han, Xin Zheng, Xueyao Wang, Tao Jin, Li Cui, Zhiguo Chen

**Affiliations:** ^1^Cell Therapy Center, Beijing Institute of Geriatrics, Xuanwu Hospital Capital Medical University, National Clinical Research Center for Geriatric Diseases, And Key Laboratory of Neurodegenerative Diseases, Ministry of Education, Beijing 100053, China; ^2^Center of Neural Injury and Repair, Beijing Institute for Brain Disorders, Beijing 100069, China; ^3^Center of Parkinson's Disease, Beijing Institute for Brain Disorders, Beijing 100069, China; ^4^Department of Neurology and Neuroscience Center, First Hospital of Jilin University, Changchun, Jilin Province, China

## Abstract

Mesenchymal stem/stromal cells (MSCs) are multipotent stem cells that can be derived from various tissues. Due to their regenerative and immunomodulatory properties, MSCs have been extensively researched and tested for treatment of different diseases/indications. One mechanism that MSCs exert functions is through the transfer of mitochondria, a key player involved in many biological processes in health and disease. Mitochondria transfer is bidirectional and has an impact on both donor and recipient cells. In this review, we discussed how MSC-mediated mitochondrial transfer may affect cellular metabolism, survival, proliferation, and differentiation; how this process influences inflammatory processes; and what is the molecular machinery that mediates mitochondrial transfer. In the end, we summarized recent advances in preclinical research and clinical trials for the treatment of stroke and spinal cord injury, through application of MSCs and/or MSC-derived mitochondria.

## 1. Introduction

Mesenchymal stem/stromal cells (MSCs) have attracted a lot of interest in basic science and clinical applications, not only due to the unique properties such as fewer ethical issues, little (if not lacking) tumorigenicity, and mild immune responses compared with other stem cell sources such as embryonic stem cells (hESCs) and induced pluripotent stem cells (iPSCs) but also because it seems to be the only stem cell type that presents both regenerative and immunomodulatory functions [1]. Engrafted MSCs can be differentiated into certain types of cells that help replenish the tissue in an autologous or allogeneic manner. In addition, MSCs show immunomodulatory properties mainly via a paracrine mechanism that involves secretion of microvesicles (MVs), microRNA, and exosomes [2, 3]. MSC-based cell replacement and immunomodulatory approaches have been employed in the treatment of some degenerative and inflammatory diseases.

Mitochondrial transfer between MSCs and damaged cells has emerged to be a promising therapeutic strategy partly because it can act as a bioenergetic supplementation [4]. Transferred mitochondria can also regulate the biological functions of cells that have taken the mitochondria (acceptor) [5, 6]. Speed and colleagues proved that mitochondria or mitochondrial DNA (mtDNA) transfer can take place between adult stem cells and somatic cells and that human lung alveolar epithelial cells harboring nonfunctional mitochondria are repaired by transfer of functional mitochondria or mtDNA from donor human bone marrow MSCs (BMSCs) [4]. This pioneer study revealed that mitochondrial donation can repair aerobic respiration in cells with dysfunctional mitochondria and protect cells from damage and apoptosis [7]. The discovery about the ability of BMSCs to transfer mitochondria to injured cells prompted a series of further studies aimed at uncovering the underlying mechanism [8–12]. Not only exerting an impact on tissues/cells in the peripheral system, mitochondrial motility is also involved in the central nervous system (CNS) diseases [13, 14], and mitochondrial transfer may open an avenue to treatment of certain neurological diseases, such as stroke and spinal cord injury (SCI). In this review, we will discuss the biological processes/outcomes at injury sites following MSC-based mitochondrial transfer and the molecular machinery required to achieve such cell-to-cell communication. In the last section, we will summarize the latest advances in therapeutic applications of MSCs and/or mitochondrial transfer to treat CNS diseases such as stroke and SCI.

## 2. Mitochondrial Transfer Impacts Cellular Metabolism and Inflammation

### 2.1. Dynamics of Mitochondria

Mitochondria are semiautonomous and self-reproducing organelles that exist in the cytoplasm of most eukaryotes [15]. Inside a cell, the number of mitochondria is regulated by two opposite processes, fusion and fission. Mitochondrial fusion process can be divided into two steps [16]: fusion of outer mitochondrial membrane (OMM) that is mediated by OMM proteins Mitofusin 1 and Mitofusin 2 (Mfn1 and Mfn2) and fusion of inner mitochondrial membrane (IMM) that is mediated by OPA1. Fission is a division event that highly depends on dynamin-related protein 1 (Drp1) to produce one or more daughter mitochondria. Drp1, together with adaptor proteins Fission 1 (Fis1), mitochondrial fission factor (MFF), and mitochondrial dynamics proteins of 49 kDa and 51 kDa (Mid49 and Mid51), are able to hydrolyze guanosine triphophate (GTP) and mediate the division of OMM and IMM. The knockdown of fusion proteins (Mfn or OPA1) or fission proteins (Drp1, Fis1, and Fis2) in MSCs disturbs otherwise a healthy mitochondria network and can even alter the stemness of MSCs [17].

Dysfunctional mitochondria are selectively degraded in a process termed “mitophagy” to maintain mitochondrial homeostasis. Activation of mitophagy in BMSCs occurs at an early stage of reactive oxygen species (ROS) stress through Jun N-terminal kinase (JNK) pathway, but declines at a late stage of ROS stress [18]. Phosphatase and tensin homolog- (PTEN-) induced kinase 1 (PINK1)/Parkin pathway, which is normally involved in the clearance of dysfunctional mitochondria [19, 20], is also required for infused MSCs to restore mitophagy pathways in hyperglycemia-challenged endothelial cells [21]. Disruption of the PINK1 pathway, and consequently the mitophagy process, may be regulated by microRNAs. MicroRNA-155 (miR-155) is one of the most prominent miRNAs detected in inflammatory and aged tissues, which directly targets B cell lymphoma-2- (Bcl-2-) associated athanogene 5 (BAG5). Reduction of BAG5 in MSCs leads to the destabilization of PINK1 and abnormality of mitophagy [22]. Also, the mitophagy process is conducive to selectively keeping healthy mitochondria and suppressing generation of ROS in MSCs, which further contributes to an immunomodulatory effect via limiting caspase-1 and interleukin-1*β* (IL-1*β*) stimulation and inhibiting inflammasome activation in macrophages [23].

### 2.2. Transferred Mitochondria Serve as a “Bioengine”

Mitochondria are known as the “powerhouse” of the cell. Each mitochondrion is surrounded by a double membrane. The inner membrane is highly invaginated, and its projection is termed cristae. Mitochondria are the source of chemical energy, generating most of the cell's adenosine triphosphate (ATP) supply via oxidative phosphorylation (OXPHOS) processes. Along with bioenergetic production, mitochondrial complexes I and III generate endogenous ROS, including oxygen radicals and hydrogen peroxide, which are involved in mitophagy and cellular apoptosis [24, 25]. The increase in ROS accumulated in normal aging or disease/injury leads to a higher rate of mitophagy and a lower level of mitochondrial biogenesis, together resulting in a reduction of mitochondrial mass [26]. Mitochondrial transfer may be able to reverse this phenomenon. For example, using an acute kidney injury mouse model, Perico et al. showed that transplantation of healthy MSCs can rejuvenate damaged tubular cells through mitochondrial transfer and restoring the energy production capacity in acceptor cells [27].

### 2.3. Mitochondrial Transfer Improves Cell Viability

Mitochondria play a critical role in cellular apoptosis [28]. ROS, a major product of mitochondria metabolism, in turn exerts a significant impact on mitochondria and mitochondria-mediated apoptosis [18]. Normally, the first stage of apoptosis involves elevated mitochondrial membrane permeability, which allows apoptogenic factors such as Bcl-2 to pass through OMM and to interrupt the electrochemical gradient in IMM. Then, the disruption of mitochondrial membrane properties results in insufficient production of ATP and activation of specific apoptogenic proteases such as caspases. Caspase-3 acts as an executor of apoptosis and activates the early steps of cellular apoptosis. Bcl-2 is able to suppress the release of cytochrome c from mitochondria via inhibiting the activation of proapoptotic factors such as Bcl-2-associated X protein (Bax) and Bcl-2-associated K protein (Bak). The imbalance of the Bax/Bcl-2 ratio is a feature that often occurs during the process of apoptosis [29]. Mitochondrial transfer from MSCs can reduce apoptosis levels and promote cell viability in recipient cells [30] via regulating the balance of Bax/Bcl-2 and reducing the expression of caspase-3 [31]. Interestingly, transfer of dysfunctional mitochondria from damaged cells to MSCs also has an influence on MSCs. Using *in vitro* and *in vivo* experiments, Gozzelino et al. showed that mitochondria released from damaged somatic cells (cardiomyocytes or endothelial cells) can be engulfed by MSCs and trigger upregulation of Heme oxygenase-1 (HO-1), a protein that protects against programmed cell death [32], and biogenesis of mitochondria in MSCs, which in turn promotes an adaptive reparative response [33].

### 2.4. Mitochondrial Transfer Promotes Anti-inflammatory Responses

**Figure 1:**
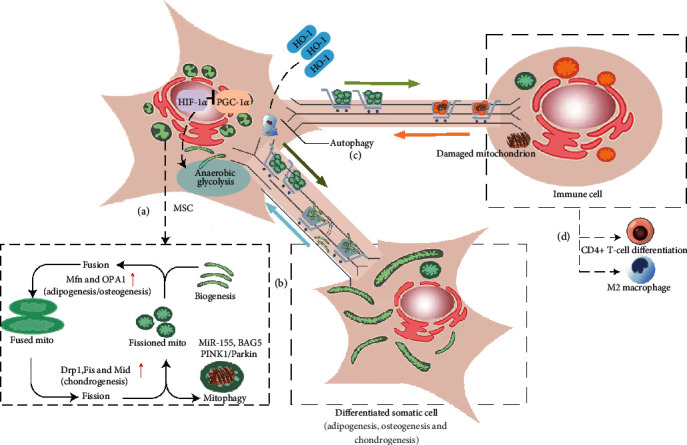
MSC-mediated mitochondrial transfer impacts cellular metabolism and differentiation. (a) Mitochondrial dynamics maintains a healthy mitochondria network in MSCs via regulating mitochondrial fusion, fission, and mitophagy. Activation of HIF1*α* under a hypoxic condition suppresses PGC1-*α* expression, leading to inhibition of mitochondrial biogenesis and the stimulation of anaerobic glycolysis. (b) The change of mitochondrial dynamics contributes to MSC differentiation and proliferation. Mitochondrial transfer may exert similar effects. (c) Somatic cell-derived damaged mitochondria are transferred and degraded in MSCs via autophagy to initiate the rescue processes; the engulfed mitochondria in MSCs lead to the upregulation of HO-1, which enhances the mitochondrial transfer capacity. (d) Mitochondrial transfer affects immune cell functions and differentiation. For example, mitochondrial transfer can suppress inflammation by promoting transition of macrophages to a M2 phenotype or inducing Treg cell differentiation.

The immunomodulatory functions of MSCs are implemented by a paracrine mechanism and cell-cell contact. The cytokines secreted by MSCs can exert a modulatory impact on various immune cells, such as T cells, B cells, natural killer cells, and macrophages [34]. It was found that mitochondrial transfer can take place between MSCs and immune cells, which influences the functions/properties of immune cells (Figure 1). Using an acute respiratory distress syndrome (ARDS) model, Krasnodembskaya's group reported that MSCs can donate mitochondria to host macrophages and enhance the phagocytic capacity and bioenergetics of macrophages, leading to an improved clearance of pathogenic bacteria [5, 35]. Along with the transfer of mitochondria, MSCs secrete exosomes containing microRNAs. After intake by macrophages, the microRNAs can target the Toll-like receptor (TLR)/NF-*κ*B pathway and dampen proinflammatory responses [36]. Nevertheless, how macrophages keep an improved phagocytotic capacity while showing a reduced proinflammatory reaction after mitochondrial transfer remains elusive. To address this issue, using an ARDS model, Morrison et al. reported that extracellular vesicle-mediated transfer of mitochondria can induce monocyte-derived macrophages (MDMs) to differentiate to an M2 phenotype with a high phagocytic capacity; and this phenotypic change mediated by mitochondrial transfer requires the OXPHOS process in macrophages [35]. In another study, Kim and Hematti cocultured MSCs with macrophages *in vitro* and found that MSCs can educate macrophages to adopt a IL-10-high, IL-12-low, IL-6-high, and tumor necrosis factor-*α*- (TNF-*α*-) low phenotype, an anti-inflammatory phenotype similar to the M2 one [37].

MSC-mediated mitochondrial transfer can also regulate T cell differentiation. Instructed by the niche cues, especially the cytokines secreted by antigen-presenting cells (APCs), T helper (CD4) cells can be activated and differentiated to various subsets, including T helper 1 (Th1), Th2, Th17, Th9, T regulatory (Treg), or T follicular helper (Tfh) cells. Among them, Th17 cells can be further divided into two subsets: proinflammatory Th17 effector cells and immunosuppressive Th17 regulatory cells. The cytokine set that drives differentiation of Th17 effector cells normally inhibits differentiation into Th17 regulatory cells, and vice versa. Luz-Crawford et al. reported that coculturing healthy donor-derived BMSCs with Th17 effector cells leads to mitochondrial transfer, which increases respiration in recipient Th17 cells and reprograms the energetic metabolism from glycolysis to OXPHOS; this change is associated with a reduced production of IL-17 and suppresses proinflammatory functions of Th17 effector cells. Interestingly, coculture with rheumatoid arthritis patient-derived BMSCs showed that mitochondrial transfer is impaired compared with that with healthy donor-derived BMSCs, suggesting that resident tissue MSCs may represent a regulatory niche to balance the proinflammatory and anti-inflammatory responses; and part of the regulatory mechanisms may be mediated by mitochondrial transfer from MSCs [38]. Similarly, a study from Court et al. demonstrated that mitochondrial transfer facilitates Treg differentiation through the enhanced expression of mRNA transcripts such as FOXP3, IL2RA, CTLA4, and TGFb1, which are involved in Treg cell differentiation [39].

Another important player in the choice-making process between Th17 effector vs. regulatory cells is hypoxia-inducible factor 1*α* (HIF1*α*). HIF1*α* and the upstream mTOR pathway are required for glycolytic activity and Th17 effector cell development, whereas deficiency in HIF1*α* leads to bias towards Th17 regulatory cell differentiation [40]. However, it is unclear how the HIF1*α* pathway may interact with mitochondrial transfer, which remains an interesting subject of future study.

Another evidence of the immunomodulatory effect is that MSCs are able to suppress airway inflammation through mitochondrial transfer to stressed epithelial cells in an asthma model. The transfer of mitochondria seems to be mediated through Miro1, a calcium-sensitive cohesive protein that can attach mitochondria to Kif5c motor protein to enhance mitochondrial transportation. MSCs that overexpress Miro1 show an improved therapeutic effect in ameliorating epithelia-mediated amplification of the immune response, through an enhanced mitochondrial donation capacity [41].

Tissue injury or degeneration is normally accompanied with inflammation, which is identified to be a driving force for mitochondrial transfer. Zhang et al. showed that the proinflammatory cytokine TNF-*α* is engaged in regulating the TNF-*α*/NF-*κ*B/TNF-*α*ip2 signalling pathway that leads to F-actin polymerization and formation of TNTs via actin-driven protrusions of cytoplasmic membrane in MSCs [42, 43]. Similarly, oxidative inflammation enhances mitochondrial transfer and increases TNT formation via the Rot/NF-*κ*B/TNF-*α*ip2 signalling pathway in a corneal wound model [11].

The impact on inflammation by mitochondrial transfer is also associated with changes in cytokine expression profiles. Lian's group reported that treatment with human iPSC-MSCs in a NADH dehydrogenase iron-sulfur protein 4 (Ndufs4) gene deficiency mouse model can protect retinal ganglion cells and reduce murine proinflammatory cytokines such as TNF-*α*, MIP-1g, GM-CSF, IL-5, IL-17, and IL-1*β* [44]. Of note, TNF-*α*, GM-CSF, MCP-1, IL-17, IL-1*β*, IL-12p70, and CD30L are closely related to NF-*κ*B signalling pathway which is involved in the regulation of TNT formation and mitochondrial transfer [45–48]. Downregulation of the above cytokines may inhibit the formation of TNTs and mitochondrial transfer. It is possible that the temporal regulation of cytokine levels correlates with the different stages of immune responses. Increased production of proinflammatory cytokines, for example TNF-*α*, may trigger the formation of TNTs and enhance mitochondrial transfer in the early phase of immune response; in the late phase of immune response, downregulation of cytokines through a paracrine mechanism by MSCs may slow down mitochondrial transfer. The results highlight the importance of applying MSCs in a right time and a right condition.

The above studies suggest that transferred mitochondria have a marked impact on immune responses via regulating macrophage and T cell functions, and through the alteration of cytokine expression. Next, we continue to discuss the impact of mitochondria biology on MSC proliferation and differentiation.

## 3. Mitochondria and MSC Proliferation and Differentiation

Mitochondrial dynamics includes the fusion and fission of mitochondria, which is crucial in maintaining the number of healthy mitochondria [49]. The morphology, distribution, density, and activity of mitochondria change along with the differentiation of MSCs to somatic terminal cells. In an undifferentiated stem cell state of MSCs, mitochondria mainly gather around the nucleus; along differentiation, mitochondria are dispersed in the cytoplasm [50, 51]. In addition, the morphology of mitochondria gradually becomes slender and elongated with well-developed cristae and an electron-dense matrix. The quantity, morphology, and distribution of the mitochondria constantly change to accommodate the energy needs which switch from a glycolysis mode at a stem cell state to an OXPHOS mode at a somatic cell state [52]. The copy number of mtDNA, protein subunits of the respiratory enzymes, oxygen consumption rate, and intracellular ATP content are all markedly increased after the induction of MSCs to osteocytes [53].

Likewise, mitochondrial transfer may influence stem cell proliferation and/or differentiation. Using a coculture system of MSCs with vascular smooth muscle cells, Vallabhaneni et al. found that mitochondrial transfer from smooth muscle cells to MSCs results in proliferation of MSCs [54]. By adding isolated normal mitochondria to iPSCs, mitochondria enter stem cells within minutes and facilitate the differentiation into neurons [55]. The evidences related to the effect of mitochondrial transfer were summarized in Table 1.

### 3.1. Mechanisms Underlying the Impact of Mitochondria Dynamics on MSC Differentiation

The morphology, quantity, and distribution of mitochondria are changed along the differentiation of MSCs. Is this change a cause or simply a consequence of differentiation? Forni et al. found that changes in mitochondria dynamics take place during the early stage of MSC differentiation; enhanced mitochondrial elongation and fusion were observed during adipogenesis and osteogenesis, and increased fission and mitophagy were observed during chondrogenesis. Knockdown of Mfn2—a factor critical in mitochondria fusion and overexpression of a dominant negative form of Drp1—a factor necessary in fission, both lead to failure of MSC differentiation, suggesting that the early changes in the mitochondria dynamics and consequently the alteration of bioenergetics are required for MSC differentiation [17].

Other factors that are closely related to mitochondrial metabolism, such as oxygen levels and ROS, may also play a role in the regulation of MSC self-renewal and differentiation. BMSCs that reside inside the bone marrow normally live in a hypoxic microenvironment, and HIF1*α* is a key regulator that can sense environment oxygen levels and adapt to it [56]. HIF1*α* pathway is activated in a hypoxic condition, which suppresses expression of peroxisome proliferator-activated receptor *γ* (PPAR*γ*) coactivator 1-*α* (PGC1-*α*), facilitates anaerobic glycolysis, and inhibits mitochondrial biogenesis [57]. MSCs cultured for a long term in a hypoxic milieu are more prone to preserve the stemness feature, as indicated by enhanced self-renewal and multipotency [58]. Compared with MSCs cultured in normoxia, hypxia leads to increased differentiation to osteocytic lineage, as exhibited by enhanced expression of markers such as osteocalcin, type I collagen, and alkaline phosphatase [59]. Meanwhile, hypoxia inhibits adipocytic differentiation, possibly by HIF1*α*-mediated suppression of PGC1-*α*, which together with the PPAR*γ* pathway promotes adipocyte differentiation [60]. The other related factor, ROS, is mainly produced by mitochondria in a cell. Accordant with the higher energy needs when MSCs differentiate to somatic cells, mitochondrial biogenesis is induced and more ROS is produced; ROS is considered to be toxic to most cellular components. To cope with elevated ROS, somatic cells usually upregulate antioxidant enzymes, which renders somatic cells more resistant to ROS than do MSCs [61]. In aged MSCs, the augmented ROS levels associated with damaged mitochondrial function may bias lineage specification towards an adipocyte fate vs. osteocyte fate [62]. It was also shown that the up-regulation of ROS can suppress osteocyte differentiation from MSCs, possibly through the inhibition of the hedgehog pathway. Furthermore, ROS seems to be necessary to initiate adipocyte specification [63], which can be inhibited by the addition of antioxidants [64]. The results suggest that oxygen levels and ROS may not simply be the consequence of differentiation but can actively influence this process. The effects and mechanisms of mitochondrial transfer between cells were shown in Figure 1.

## 4. Factors That Affect Mitochondrial Transfer

### 4.1. “Machinery” for Mitochondrial Transfer

Intercellular mitochondrial transfer involves three steps. First, specific signals are required from the damaged cells and/or other niche factors to trigger the process; second, the machinery/structure is formed to facilitate the transfer; and third, mitochondria are transported and perform certain functions in the receptor cells.

Using an ischemic cellular model, Liu et al. reported that phosphatidylserines exposed on the apoptotic endothelial cells can trigger mitochondrial transfer from infused MSCs to rescue the respiration functions of endothelial cells [6]. Secreted mitochondria released from damaged cells may also act as a “danger signal” to trigger mitochondrial donation [33, 65]. Specifically, somatic cell-derived mitochondria are transferred and degraded inside MSCs to initiate the rescue processes. It would not be surprising that other initiating signals also exist and exert functions in different settings.

Different molecular structures have been reported that mediate intercellular mitochondrial transfer, including tunneling nanotubes (TNTs), gap junction, extracellular vesicles (EVs), free extracellular mitochondria, and cytoplasmic fusion [66, 67]. Due to the limit on the scope of this review, we will mainly focus on the formation of TNTs and gap junction. For a more comprehensive review on this subject, please refer to these articles [68, 69]. TNTs are identified as a nanotube that can transport proteins, lipid droplets, ions, RNAs (including mircoRNAs), organelles, viruses, and cytosol in both directions [70]. Membrane-bound proteins were also observed to be transported between cells via TNTs [71]. MSCs are often used in coculture systems to study the function of TNTs, which can be formed over “long” distances (150 mm) when cells are far away from each other [71]. Two types of TNTs have been observed between human monocyte-derived macrophages, thin TNT and thicker TNT, which can be distinguished by their cytoskeleton structure, size, and functional characteristics [72]. Thicker nanotubes are longer, larger (600-700 nm in diameter) channels that contain microfilaments, microtubules, and F-actin, whereas thin-membrane nanotubes normally only contain F-actin. Most of mitochondrial transfer and intracellular vesicles transfer, but not all, seem to take place within thick-membrane TNTs between macrophages. As for some other types of cells, for example, kidney cells and neurons, TNTs formed between cells seem to mainly contain F-actin, but not microtubules [73]. Rustom and colleagues showed that multiple TNTs could form between cells, forming a complex 3-D network [70]. It is possible that the types of TNTs and the cargoes transported would vary between different cell types. In addition to transportation via TNTs, Li and colleagues revealed that gap junction is also involved in mitochondrial transfer from BMSCs to motor neurons [74]. In certain context, the tip of the nanotube can be embedded with gap junction proteins that are juxtaposed to the other gap junction proteins in the membrane of receptor cells. The gap junctions may facilitate mitochondrial transfer and allow electrical coupling between distant cells, which may represent another important means of intercellular signalling [75].

### 4.2. Origin and Status of MSCs Affect Mitochondrial Transfer

Several factors impact the formation of TNTs and further influence the efficiency of mitochondrial transfer. Motor protein, Kif5c, enables mitochondria to transfer along the microtubule network [76, 77]. Miro1 (mitochondrial Rho-GTPase), a calcium-sensitive cohesive protein, with the help of accessory proteins such as Miro2, TRAK1, TRAK2 and Myo19, can associate the mitochondria to Kif5c motor protein and assist the mitochondria to move along microtubules [78, 79]. Bioengineered MSCs that overexpress Miro1 show increased mitochondrial transfer to injured epithelial cells and a greater reparative capacity, while knockdown of Miro1 results in loss of reparative effect [41]. PINK1 and Parkin target Miro for degradation and thus can arrest mitochondrial mobility [80]. In addition, shRNA-mediated knockdown of CD38 [81] and TNF-*α* [82] inhibits TNT formation and blocks mitochondrial transfer *in vitro*. Connexin 43 (CX43) is a gap junction protein. In an allergic airway inflammation model, Yao et al. showed that the overexpression of CX43 enhances the rescue efficacy of mitochondrial dysfunction and allergic inflammation, while silencing of CX43 partially nullifies this protective effect [83]. Apart from the factors that directly affect the formation of TNTs, hypoxia/reoxygenation [31], inflammatory stresses [11], and chemotherapy stress [84] may indirectly stimulate TNT formation. Besides, the microenvironment is a significant factor that regulates mitochondrial transfer. A study from Zhang's group suggested that the proinflammatory microenvironment is critical to provoke mitochondrial transfer from iPSC-MSCs to damaged cardiomyocytes [42]. NADPH oxidase 2- (NOX2-) derived superoxide in distressed cells stimulates ROS generation in BMSCs, which further leads to increased mitochondrial donation from BMSCs [85]. Oxygen-glucose deprivation (OGD) treatment on astrocytes or pheochromocytoma (PC12) cells promotes mitochondrial transfer from MSCs [82]. In addition, several factors that affect mitochondrial biogenesis or dynamics can enhance the process of mitochondrial transfer, such as HO-1, OPA1, and KD (mitochondrial fusion protein knockdown). The above results indicate that manipulation of the transfer machinery and/or the microenviroment may offer an effective approach to further enhance the efficiency and extent of mitochondrial transfer.

The origins and cellular states of donor cells also impact mitochondrial transfer. MSCs can be obtained from various tissues or differentiated from pluripotent stem cells. MSCs isolated from different tissues such as the bone marrow (BM), adipose (AD), dental pulp (DP), and Wharton's jelly (WJ) display differential mitochondrial donation capacity and therapeutic effects [86]. WJ-MSCs and DP-MSCs, compared with AD-MSCs and BM-MSCs, show higher respiratory capacity and bioenergetics and achieve a better rescue effect in damaged cardiomyocytes with a relatively smaller number of transferred mitochondria [86]. Moreover, compared with BM-MSCs, iPSC-derived MSCs (iPSC-MSCs) show superior effects in a limb ischemia model [45] and exhibit a higher efficiency of mitochondrial transfer to stressed cells in a chronic obstructive pulmonary disease model [87] and an anthracycline-induced cardiomyopathy model [42]. The greater ability of mitochondrial transfer in iPSC-MSCs could be attributed to a higher expression level of Miro1 and TNF-*α*IP2 [42]. Mitochondrial transfer from iPSC-MSCs was also shown to be beneficial in CoCl-insulted pheochromocytoma cells (PC12) [88] and cigarette smoke-exposed airway cells [89]. Interestingly, the beneficial effects of iPSC-MSCs on damaged cells may not only be entirely attributable to mitochondrial transfer but also to paracrine effects. iPSC-MSCs vs. bone marrow- or cord-derived MSCs are enriched with certain cytokines. For example, macrophage migration inhibitory factor (MIF) and growth differentiation factor-15 (GDF-15) are uniquely released from iPSC-MSCs to account for a cardioprotective effect, which is independent of mitochondrial transfer [90]. Notably, MSCs show beneficial effects through paracrine actions in cardiac repair [91] and hypoxia-conditioned media contain a higher expression of several growth factors that further promote the cardioprotective effects [92].

The cellular state of MSCs is also an important factor affecting the efficiency of mitochondrial transfer. By comparing the efficacy in a corneal wound healing experiment between healthy iPSC-MSCs and Rotenone-treated iPSC-MSCs, Jiang et al. pointed out that only healthy iPSC-MSCs display a beneficial effect [11]. Compared with Rotenone-treated iPSC-MSCs, healthy iPSC-MSCs show a higher level of basal mitochondrial oxygen consumption rate, ATP production, and maximal respiration. MSCs with impaired mitochondria (i.e., aged MSCs) may not be suitable therapeutic donors as only healthy functional mitochondria could fully exert the protective effects [11, 42]. Furthermore, the cell types that mitochondria are derived from also impact the outcome. Court et al. showed that exogenous mitochondria freshly isolated from MSCs can induce T cells to adopt a Treg phenotype; but this effect is not achieved by mitochondria isolated from other cell types such as fibroblasts or peripheral blood mononuclear cells, stressing the importance of the source of mitochondria [39].

In short, successful mitochondrial transfer requires sophisticated orchestration of several processes/signals, such as initiating signals, formation of transfer structure, and regulatory factors to control the speed of transfer. Besides, the significance of the source and status of mitochondrial donor cells should not be underestimated. Next, we will discuss the application of mitochondrial transfer in treatment of some neurological diseases.

## 5. Mitochondria-Based Therapy in Treatment of Neurological Diseases

Mitochondrial dysfunction is associated with various neurological pathologies, and transferring healthy mitochondria may be a new approach to restore mitochondrial functions [13, 14]. Mitochondrial transfer can be used to correct a range of problems caused by mitochondrial dysfunction via activating metabolic or immunomodulatory signalling pathways. In addition, cellular transfer of mitochondria is accompanied with the horizontal transfer of mitochondrial genes. Thus, genetically normal or enhanced mitochondria could be introduced to treat mitochondrial gene-related diseases (this topic is not discussed in this article due to scope limit). Below, we will summarize recent advances in mitochondria-based treatment on two common neurological diseases, stroke and SCI.

### 5.1. Mitochondria-Based Therapeutics for Treatment of Stroke

Acute ischemic stroke (AIS) occurs when the artery/arteries supplying the brain are blocked. The reduced blood flow results in cellular dysfunction, damage, and/or death, which underscores the importance of rapid blood flow recovery. Although revascularization is desired for stroke treatment [93], transport of oxygen and nutrient to the damaged tissues often leads to the activation of the innate and adaptive immune responses that may cause secondary damage to the remaining cells [94, 95].

Mitochondrial dysfunction has been recognized as a hallmark in the complex cellular processes of ischemia/reperfusion (I/R) injury, which is characterized by reduced ATP production, increased ROS production, and elevated cell death [6]. When the blood supply is reduced or absent during ischemia, cells switch to anaerobic glycolytic metabolism, which gives rise to accumulation of lactic acid, H^+^, NADH^+^, and a lower level of ATP production. Consequently, Ca^2+^ reuptake from cytosol is impaired and additional Ca^2+^ influx is promoted by reperfusion, together leading to Ca^2+^ overload in cells [96]. A high level of Ca2^+^ and oxidative stress result in the opening of mitochondrial permeability transition pore (MPTP) in the inner mitochondrial membrane and mitochondrial membrane uncoupling, which further augments ROS production [97, 98]. The excessive ROS production may cause damage on protein, DNA, and lipid, eventually leading to cell death [99, 100]. On the other hand, ROS can also induce astrogliosis [101], and chronic astrogliosis may impede regeneration of neural tissues [102]. Interestingly, although previous studies suggested that ROS and calcium participate in a viscous cycle of tissue damage, the latest research indicates that calcium may not stimulate the production of free radicals but suppress them [103].

To cope with the pathological damage caused by mitochondrial dysfunction in ischemia-reperfusion injury, mitochondrial transfer may be beneficial. Sources of mitochondrial transfer include astrocytes, endothelial cells, and MSCs. In brain, neurons and astrocytes can exchange mitochondria. Damaged mitochondria are released from neurons and taken by astrocytes for disposal and recycling. In a transient focal ischemia model, Lo's group found that astrocytic mitochondria are released and taken by injured neurons as a protective mechanism; and the process is mediated via a calcium-dependent mechanism involving CD38 and cyclic ADP ribose signalling [104]. Extracellular mitochondria collected from astrocytes, when injected into the peri-infarct area of a focal cerebral ischemia mouse model, can be taken by the neurons [104], suggesting that mitochondrial injection may be a novel therapeutic approach to treat stroke. In a follow-up study, the authors reported that free mitochondria exist in the cerebrospinal fluid in subarachnoid hemorrhage patients, and the membrane potentials of the mitochondria correlate with the clinical outcomes three months after stroke [105].

In stroke, not only neurons but also the neurovascular units are damaged, which include neurons, astrocytes, endothelia, and pericytes. Lo's group also pioneered in investigating the effect of mitochondria secreted from endothelial progenitor cells (EPCs) in an OGD model [106]. EPCs exist in circulating blood and are capable of homing to damaged areas to promote vasculogenesis. Addition of EPC-derived mitochondria into OGD-injured brain endothelium can restore endothelial tightness, promote angiogenesis, and increase intracellular ATP levels [106].

The most often used source of mitochondrial transfer is MSCs. Coculture of MSCs with OGD-treated human umbilical vein endothelial cells results in mitochondrial transfer to the damaged cells, and the process is initiated by recognition of the phosphatidylserines exposed on the surface of apoptotic endothelial cells. Using a middle cerebral artery occlusion (MCAO) and reperfusion rat model, Li et al. found that MSCs engrafted into the damaged area can donate mitochondria to the injured cerebral microvasculature [87]. Due to the ease of access, low immunogenicity, and good safety, MSCs are currently being trialled in stroke patients. On the website of ClinicalTrials.gov, as of the writing of this review, more than 20 clinical trials have been registered in which MSCs are applied to treat stroke patients. The MSCs used were derived from different sources such as the bone marrow, adipose, and umbilical cord, as either autologous or allogeneic graft, and the locations of those trials include various countries such as the United States, China, South Korea, and Spain. The delivery routes and the types of strokes selected also vary across trials. The extensive clinical trials hold great promise for the development of new MSC-based therapeutic drugs and/or approaches to treat stroke.

### 5.2. Therapeutics for the Treatment of Spinal Cord Injury

SCI, normally resulting from traumatic external forces, is categorized into two stages—primary injury and secondary injury [107]. During the secondary injury, ruptured blood vessel and reflexive vasoconstriction that result from the acute spinal cord injury may lead to a reduction in oxygen delivery and consequently damage those oxygen-dependent organelles such as mitochondria. The impaired mitochondria are less capable of maintaining its homeostasis and dynamics, resulting in energy insufficiency [108]. Secondary damage in SCI also comprises a cascade of events that trigger additional pathologies, such as mitochondrial permeability damage, calcium overload, excitatory toxicity, oxidative stress, and increased ROS production [109]. Different approaches such as repairing or replacing damaged mitochondria (mitochondrial transplantation), introduction of alternate energy sources (“biofuels”), use of antioxidant, and restoring mitochondrial permeability are currently being contemplated to deal with the second injury in SCI [110].

Mitochondrial transplantation, either of endogenous or exogenous origin, has shown encouraging outcomes in the replacement of dysfunctional mitochondria [111]. Recently, exogenous mitochondria isolated from PC12 cell line or rat muscle tissues were transplanted into injured rat spinal cord and observed to restore energy supply to injured tissues. Unfortunately, these transplanted mitochondria failed to produce long-term (6 weeks after injury) functional neuroprotective effects [112]. The reason for the mild long-term efficacy was not fully understood, but one possibility may lie in the cellular source of engrafted mitochondria. In a separate study, Li et al. injected either MSCs or MSC-derived mitochondria into the injured spinal cord of a rat contusion SCI model and observed significantly improved locomotor functions 6 weeks after injury [74]. Further studies are needed to compare the efficacy of mitochondria isolated from different sources in the same experimental setting. The secondary injury in SCI consists of many different aspects, such as inflammation, damaged bioenergetics, and inhibitory niche for axonal regrowth, and addressing any single aspect by a particular approach may not be sufficient to amount to a dramatic interventional effect [109, 110, 113]. The multifactorial properties of MSCs may be advantageous in this regard. MSCs can regulate inflammatory responses, have a good capacity to donate mitochondria, and are able to secrete trophic factors; these properties may underlie the popular use of MSCs for treatment of different indications that include SCI [114, 115]. *In vitro* and *in vivo* studies showed that MSCs seem to be able to alleviate the secondary injury caused by inflammation [116], restore myelin insulation, promote axonal regeneration, and assist in angiogenesis [117–121]. Sykova et al. reported that the survival and efficacy of MSC graft can be enhanced by cotransplantation of appropriate biomaterials [122, 123]. In this study, Sykova and colleagues also tested intravenous and intraarterial delivery of MSCs in 20 SCI patients and confirmed the safety of this approach [122, 123]. Deng and colleagues conducted a phase I clinical trial by engrafting umbilical cord-derived MSCs with collagen biomaterial in 20 SCI patients (acute complete cervical injury), with the other 20 patients (acute complete cervical injury) who received biomaterial only as the control group. After a 12-month follow-up, the treatment group vs. control group showed significantly improved American Spinal Injury Association scores and better bowel and urinary functions [124]. In earlier clinical trials in which MSCs were applied to treat SCI patients, some clinical benefits were also observed [125–127]. Nevertheless, larger patient cohorts and randomized double-blind trials are necessary to draw a firm conclusion on the efficacy of this approach. At present, more than 30 clinical trials using MSCs for SCI treatment have been registered at ClinicalTrials.gov. In the coming years ahead, we will for sure see more data on the clinical efficacy of various types of MSCs on different types of SCIs. However, in those trials, it is the live MSCs that are applied for treatment of SCI; yet, no MSC-derived extracellular mitochondria have been tested in clinical trials. With the fast advance of the field and more consolidating preclinical data emerging on the efficacy of mitochondrial engraftment, clinical trials that involve transplantation of mitochondria alone or in combination with other effectors are warranted in the future.

## 6. Limitation and Future Perspectives

The ability of mitochondria to be transferred between cells has attracted a lot of attention in the past decades and an increasingly larger body of literature are emerging to unravel the detailed mechanisms of this phenomenon. However, there are still many open questions existing in the field which require further studies.

(1) MSCs are used as a popular donor of mitochondria in many studies that mostly focused on the transfer of mitochondria from MSCs to damaged cells. The transfer of mitochondria is actually a “two-way” transportation, and it is still unclear under what conditions would one way dominate the other and how this directionality of transport is initiated and regulated. (2) Different means of mitochondrial transfer have been reported that include TNTs, gap junctions, microvesicles, free extracellular secretion, and cell fusion. Can cells use multiple ways to transfer mitochondria at the same time? Is the choice of means cell type specific and/or microenvironment dependent? If so, how is this decision-making process determined and regulated? (3) To what extent is mitochondrial transfer participating in cellular repair as an intrinsic mechanism in organisms and to what extent following exogenous transplantation in disease? Is there any way to manipulate the extent of mitochondrial transfer to be clinically meaningful or to further increase the clinical efficacy? (4) Mitochondrial dynamics is regulated by both mitochondrial genome and nuclear genome. An indepth understanding of the regulatory mechanisms would definitely facilitate designs of small molecules, gene editing approaches, and other novel strategies, to improve the health state of mitochondria and the capacity to donate. (5) Compared to MSCs, do mitochondria derived from other cellular sources, such as astrocytes, endothelial cells, induced neural stem cells, and induced pluripotent stem cells, differ in the properties and therapeutic effects? (6) Will allogeneic or exogenic mitochondria be recognized by host immune system after engraftment? Would immune disparity still play a role after the uptake of exogenous mitochondria or even after the incoming mitochondria having fused with host mitochondria? (7) How to solve the scale-up issue if mitochondrial transfer proves to be an efficacious and safe therapy in the future? Can immortalized or genetically enhanced MSCs produce equally safe and efficacious mitochondria? (8) Mitochondrial transfer may be beneficial to damaged somatic cells in certain context but may be deleterious in other cases. Mitochondria transferred to cancer cells could enhance the bioenergetics of cancer cells and increase the invasiveness and resistance to drug treatment. In these cases, suppression of mitochondrial transfer may be desired.

With a deep understanding of the detailed mechanisms of mitochondrial transfer and extensive preclinical investigation on various disease models, it is not unrealistic to predict that the gap between basic research and clinical application may be closed in the foreseeable future.

## 7. Conclusion

Mitochondrial transfer is considered a promising therapeutic strategy, not only because it can restore mitochondria-related metabolism in damaged cells but also due to the ability to regulate many other basic aspects of a cell, such as cell survival, proliferation, and differentiation. Development of regenerative medicine that involves mitochondrial transfer offers a great potential for the treatment of neurological diseases such as stroke and spinal cord injury.

## Figures and Tables

**Table 1:** Evidences of mitochondrial transfer between cells.

Donor cells	Acceptor cells	Methods	Mitochondrial transfer manner	Cell fate	Biological outcome	Related mechanisms	References
RL14 or HUVEC	MSCs	Coculture (MSCs + damaged cells); exposure of MSCs to exogenous somatic mitochondria	Bidirectional	Enhance MSCs viability	Upregulation of HO-1, PGC-1*α*, and mtTFA stimulates mitochondrial biogenesis in MSCs; HO-1 promotes mitochondrial transfer from MSCs to damaged cells	Somatic-derived mitochondria are engulfed and degraded by MSCs to trigger mitochondrial transfer from MSCs to damaged cells; elevated ROS upregulates HO-1; HO-1 enhances antiapoptotic function of MSCs and damaged cells; increase expression of Miro-1	[33]

MSCs	Fibroblasts	Coculture (MSCs+ fibroblasts+ OPA1 KO mouse fibroblasts); TNF-*α* is added	Bidirectional; mitochondrial transfer via TNTs and cellular fusion; promote mitochondrial transfer by TNF-*α* or 2DG treatment and OPA1 knockout	Increase mitochondrial biogenesis	Reduce ROS and improve redox homeostasis	Mitochondrial transfer reduces ROS but fails to salvage CI deficiency	[128]

BMSCs	PTECs	Coculture (BMSCs+ PTECs); *in vivo* (rat model)	Intravenously administered BMSC-derivedisolated mitochondria to PTECs	Enhance cell viability; recover the expression of Megalin and SGLT2; reorganize tubular epithelium	Inhibit ROS production	Incorporated Mt acts on the endogenous Mt of PTECs, which suppresses cellular apoptosis via regulating Bcl-2, Bax, and PGC-1*α*; increase SOD2 and reduce ROS production	[30]

BMSCs	RCNs	Coculture (BMSCs+ RCNs); *in vivo* (injected cocultured cells in rats)	Unidirectional; elevate expression of Miro1	Fail to detect the expression of neurospecific *β*-III-tubulin or GFAP	BDNF	—	[129]

MSCs	Astrocytes and PC12 cells	Coculture (MSCs+ astrocytes exposed to OGD/PC12 cells); *in vivo* (rat models)	Unidirectional; mitochondria transfer via TNTs	Stimulation of neural cell proliferation	Restore respiration; show neuroprotective effect	Overexpression of Miro1 promotes mitochondrial transfer	[82]

VSMCs	BMSCs	Coculture system	Unidirectional; mitochondrial transfer via TNTs; formation of thin TNT-like structures	Fail to induce MSC differentiation to VSMC-like phenotype but successfully induce MSC proliferation	Stressed cells with dysfunctional mitochondria can trigger mitochondrial transfer	—	[54]

BMSCs	AML cells	Coculture (BMSCs+ AML or non-malignant CD34^+^); *in vivo* (inject primary AML blasts without NOX2+ BMSCs)	Unidirectional; mitochondrial transfer mainly via TNTs, and to a small extent through endocytosis	Enhance cell viability and proliferation in AML cells; increase mitochondrial biogenesis	Increase mitochondrial respiration; mitochondrial transfer promotes disease progression	NOX2-generated superoxide stimulates ROS production in BMSC; ROS enhances mitochondrial transfer to leukemic blasts	[85]

BMSCs	Mouse melanoma and breast carcinoma cells derived from cells	*In vivo* (mouse)	mtDNA transfer	Stimulation of tumor cell proliferation	Delay tumor initiation; restore mitochondrial respiration in tumor cells via recovering respirasome and CII	mtDNA acquisition recovers mtDNA transcription and restores mitochondrial protein synthesis	[130]

MSCs	CD4+ T cells	Coculture (BMSCs/RA-MSCs^8^+Th17 cells)	—	Induce Treg and suppress Th17 differentiation	Immunomodulation	—	[38]

hMADs	Cardiomyocytes	Coculture (hMADs+ cardiomyocytes)	Unidirectional; mitochondrial transfer via cell fusion and TNTs	Reprogram adult cardiomyocytes towards a progenitor-like state	—	Mitochondrial transfer and partial fusion between hMAD and cardiomyocytes reprogram cardiomyocytes to a cardiac progenitor-like state	[131]

MSCs	MDMs	Coculture (MSCs +MDMs); isolation of MSC-derived EVs; *in vivo* (LPS-induced lung injury model)	Extracellular vesicles (EVs)	Coculture with MSCs increases the percentage of MDMs expressing CD206	Promote oxidative phosphorylation and enhance anti-inflammatory and phagocytic effect	LPS treatment stimulates MDM secretion of M1 associated chemokines, TNF-*α* and IL-8, and M2 chemokines CCL18 and CCL22; both chemokines are diminished by addition of MSCs; MSCs show anti-inflammatory effect and enhance phagocytosis that can be attributed to MSC-derived EVs expressing CD44	[35]

MSCs	T cells	Coculture (MSCs+T cells);	Unidirectional;	Induce Treg and suppress Th17 differentiation	Immunomodulation	Mitochondrial transfer from MSCs drive Treg differentiation (CD25^+^FoxP3^+^) via overexpression of mRNA transcripts (FOXP3+, CTLA4,IL-2RA, and TGF-b1)	[39]

iPSC-MSCs	CMs	Coculture (iPSC-MSCs+CMs)	Bidirectional; mitochondrial transfer via TNTs	—	Augment mitochondrial retention and bioenergetic reservation	TNF-*α* is engaged in regulating TNF-*α*/NF-*κ*B/TNF-*α*ip2 signalling pathway which is able to enhance the formation of TNT	[42]

MSCs	CECs	Coculture (MSCs+CECs); *in viv*o (alkali-injured eyes in a rabbit model)	Mitochondrial transfer via TNTs	—	Corneal protection	ROS activates NF-*κ*B in CECs and enhances TNT formation via upregulation of NF-*κB*/TNF-*α*ip2 signalling pathway	[11]

iPSC-MSCs	ASMCs	Coculture (iPSC-MSCs+ASMCs); *in vivo* (an ozone-induced mouse model of COPD)	Unidirectional; mitochondrial transfer via TNTs	—	Attenuate ozone-induced mitochondrial dysfunction, airway hyperresponsiveness and inflammation through mitochondrial transfer and paracrine effects	The protective effect may be exerted through mitochondrial transfer and paracrine effects	[89]

iPSC-MSCs	PC12 cells	Coculture (iPSC-MSCs+PC12 cells)	Unidirectional; mitochondrial transfer via TNTs	—	Prevent apoptosis, mitochondrial swelling, and restore ∆*Ψ*m in damaged cells	—	[88]

iPSC-MSCs	RGCs	Coculture (iPSC-MSCs+ RGCs); *in vivo* (transplanted iPSC-MSCs into the retina of Ndufs4 KO mice)	Unidirectional	—	Reduce abnormal activation of glial cells and neuroinflammation	Paracrine action and mitochondrial transfer are an interaction of two independent processes in MSC-mediated cell protection	[44]

Note: AML: acute myeloid leukemia; ASMCs: airway smooth muscle cells; Bax: Bcl-2 associated X protein; Bcl-2: B cell lymphoma-2; BDNF: brain-derived neurotrophic factor; BMSCs: bone marrow mesenchymal stem cells; CCL18: chemokine cc motif ligand 18; CCL22: chemokine cc motif ligand 22; CECs: corneal epithelial cells; CI: mitochondrial complex I; CII: mitochondrial complex II; CMs: cardiomyocytes; COPD: chronic obstructive pulmonary disease; GFAP: glial fibrillary acidic portein; hMADs: human multipotent adipose-derived stem cells; HO-1: heme oxygenase-1; HUVECs: human umbilical vein endothelial cell; IL-8: interleukin-8; LPS: lipopolysaccharide; MDMs: monocyte-derived macrophage; Miro 1: mitochondrial Rho-GTPase 1; MSCs: mesenchymal stem cells; mtDNA: mitochondrial DNA; mtTFA: mitochondrial transcription factor A; *Ndufs 4*: NADH dehydrogenase (ubiquinone) Fe-S protein 4; NF-*κ*B: nuclear factor-kappa B; NOX2: NADPH oxidase 2; OGD: oxygen-glucose deprivation; OPA1: the mitochondrial inner membrane fusion protein optic atrophy 1; PC12 cells: pheochromocytoma cells; PGC-1*α*: PPAR*γ* coactivator 1*α*; PTECs: proximal tubular epithelial cells; RA-sMSCs: rheumatoid arthritis synovial stromal stem cell; RCNs: rat cortical neurons; RGC: retinal ganglion cell; ROS: reactive oxidative stress; SOD2: superoxide dismutase 2; SGLT2: sodium-glucose cotransporter; TNF-*α*: tumor necrosis factor *α*; TNTs: tunneling nanotubes; Treg: T regulatory cells; VSMCs: vascular smooth muscle cells; 2DG: a glucose analogue that inhibits glycolysis, thereby reducing glycolytic flux; *∆Ψ*m: mitochondrial membrane potential.
